# PD60 Leveraging Real-World Evidence For Rare Disease And Oncology Health Technology Assessments: Insights From Pulse Infoframe Inc.’s Hybrid Approach

**DOI:** 10.1017/S026646232510192X

**Published:** 2025-12-29

**Authors:** Daniel Lewi, Femida Gwadry-Sridhar

## Abstract

**Introduction:**

Health technology assessment (HTA) of rare diseases and cancer face significant challenges in illustrating value. This is compounded by small patient populations, limited data, and undefined endpoints. We tackled these barriers by engaging patients directly, defining a pragmatic data strategy, combining site-based and decentralized models focusing on patient-first data collection, and incorporating patient preferences to generate high quality real-world evidence (RWE) to inform HTA decisions.

**Methods:**

A relevant use case supporting pricing reimbursement for a uveal melanoma treatment required ambispective data that illustrated an unmet need. Integrated data, including patient-reported outcomes and clinical registries can help shape better therapeutic practices and inform regulatory approval pathways. This can then support data capture and inform patient-centric endpoints for regulatory approval, aligning clinical trials with real-world needs.

**Results:**

The data collected in a RWE platform and used in conjunction with early access program data resulted in pricing reimbursement in multiple regions. Substantiating unmet need requires robust, long-term data collected in a standardized data format in the Pulse registry platform to demonstrate disease progression in untreated patients. A similar strategy was used in lung cancer to support a pricing dossier and subsequent approval. The design of studies relevant for HTA requires a data driven approach with relevant and pragmatic study design that focuses on expected patient benefits. These examples were conducted on historical cohorts but should include contemporaneous data and patient input through preferences and outcomes in future analyses.

**Conclusions:**

The road to accessing therapies is not a straight line. Our work demonstrated how RWE bridged gaps between clinical trials and real-world applications. By aligning patient needs with regulatory priorities, RWE facilitates informed, patient-centered decisions, improving access to lifesaving therapies for patients with rare diseases or cancer worldwide.

